# Screening of core filter layer for the development of respiratory mask to combat COVID-19

**DOI:** 10.1038/s41598-021-89503-x

**Published:** 2021-05-13

**Authors:** Lokesh K. Pandey, Virendra V. Singh, Pushpendra K. Sharma, Damayanti Meher, Utpal Biswas, Manisha Sathe, Kumaran Ganesan, Vikas B. Thakare, Kavita Agarwal

**Affiliations:** 1 Defence Research and Development Establishment, DRDO, Jhansi Road, Gwalior, 474002 India; 2 Defence Materials and Stores Research and Development Establishment, Kanpur, 208013 India

**Keywords:** Diseases, Risk factors

## Abstract

The severe outbreak of respiratory coronavirus disease 2019 has increased the significant demand of respiratory mask and its use become ubiquitous worldwide to control this unprecedented respiratory pandemic. The performance of a respiratory mask depends on the efficiency of the filter layer which is mostly made of polypropylene melt blown non-woven (PP-MB-NW). So far, very limited characterization data are available for the PPE-MB-NW in terms to achieve desired particulate filtration efficiency (PFE) against 0.3 µm size, which are imperative in order to facilitate the right selection of PP-MB-NW fabric for the development of mask. In present study, eight different kinds of PP-MB-NW fabrics (Sample A–H) of varied structural morphology are chosen. The different PP-MB-NW were characterized for its pore size and distribution by mercury porosimeter and BET surface area analyzer was explored first time to understand the importance of blind pore in PFE. The PP-MB-NW samples were characterized using scanning electron microscopy so as to know the surface morphology. The filtration efficiency, pressure drop and breathing resistance of various PP-MB-NW fabric samples are investigated in single and double layers combination against the particle size of 0.3, 0.5 and 1 µm. The samples which are having low pore dia, high solid fraction volume, and low air permeability has high filtration efficiency (> 90%) against 0.3 µm particle with high pressure drop (16.3–21.3 mm WC) and breathing resistance (1.42–1.92 mbar) when compared to rest of the samples. This study will pave the way for the judicial selection of right kind of filter layer i.e., PP-MB-NW fabric for the development of mask and it will be greatly helpful in manufacturing of mask in this present pandemic with desired PFE indicating considerable promise for defense against respiratory pandemic.

## Introduction

A new outbreak of corona virus disease (COVID-19), a viral contagious infection has engulfed many life globally and remain persist as major global threat to the human beings^1^. The disease is caused by infection with the severe acute respiratory syndrome coronavirus 2 (SARS-CoV-2) and primary transmission is through virus-filled respiratory aerosol droplets and can be produced by an infected person when coughs, sneezes, speaks or breathes, though specific mode of transmission is still debatable^2–5^. These aerosol droplets are having a different aerodynamic sizes, the droplet which has aerodynamic diameter more than 5 µm settles due to gravity near the source and hence, it reduces the chances of spread, however the fine aerosol particle having aerodynamic size less than 5 µm remain suspended in the environment for longer duration and play a key role for the spreading of infection^6^. Though the ultimate fate of aerosol particles are greatly influenced by the various environmental factors such as humidity, temperature, etc. which determine the virulence of aerosol particle^2^.

In the past also world has witnessed many deadliest outbreak including cholera, Spanish flu, Ebola etc. and now corona outbreak has raised a serious threat for human life and emerged as a major challenge for the scientific fraternity to control its widespread infection^7^. The World Health Organization (WHO) on March 11, 2020 declared that the COVID-19 is a pandemic and had endangered more than 218 countries and territories affecting more than 6.4 crore human life around the world and the sustained risk of further global spread^8^. Government regulatory authorities are working on the World Health Organization’s (WHO) guidelines and has taken a protective measure to prevent this world disaster unleash spread of deadliest corona pandemic by advancement in sanitization and public hygiene and take a serious measures in terms of isolation, quarantines etc. along with the use of appropriate personal protective equipment for healthcare workers in practice^9,10^. Amidst the lack of vaccine against this COVID-19, personal hygiene and use of facemask have been suggested as important mitigation strategies against this type of respiratory infection, however, uncertainties exist for the mode of transmission of COVID-19^11,12^. In the wake of the COVID-19 pandemic, the US center for disease control and prevention recommends the use of face masks as a physical barrier in public places to prevent its onward transmission^13^. The purpose of physical respiratory protection is to limit the exposure of individuals against particular threat and it works on twin aspects of protection (i) establishment of artificial barrier: filter layer (melt blown) and (ii) supply of breathable air. In this COVID-19 pandemic, respiratory masks core layer i.e. filter layer significantly improves the protection of front line fighters in mitigating this spread of coronavirus via respiratory droplets^14,15^. These airborne aerosol particles are trapped by filter layer of mask through a combination of the following mechanisms: Interception, Sedimentation, Impaction and Diffusion^16^. In general, the efficiency of the aerosol retention depends on the fiber diameter of the medium, particle size of the aerosols, and rate of airflow through the filter^17^. The filtering facepiece respirators (FFRs) as per the US National Institute for Occupational Safety and Health (NIOSH) classifies particulate into various categories (N95, N99, N100, P95, P99, P100, R95, R99, and R100)^18^ where, N refers that the respirators is not resistant to oil droplet; R refers resistant to oil droplet, P is completely resistant to oily aerosols and numerical digit indicates the percentage minimum filtration efficiency^19^. However, BIS standard (IS 9473:2002) classifies filtering half mask into three classes: FFP1, FFP2, and FFP3 with corresponding minimum filtration efficiencies of 80%, 94%, and 97%^20^. The lock down due to COVID-19 severely affects the production and supply of face masks throughout the world^21^ and its worldwide shortage during the COVID-19 outbreak has become a social concern^21,22^. Many industries which were not involved in this business have also entered in to this sector for fulfilling the scarcity of mask in the market. However, due to the lack of judicial selection of filter material of mask, design, inadequate knowledge of different layers of mask, impeded the fast development and production of facemask by the start-up industries. In order to ensure the availability of mask during the COVID-19 outbreak, the knowledge about the different component of facemask is very critical to intensify efforts to develop effective facemask which ensure the highest possible user comfort along with high PFE^22^. In facemask, filter layer is the vital component and plays a very important role for determining the filtration efficiency^22^. Filter layer can be made up of different material starting from polypropylene and polyethylene melt blown non woven/woven/knitted, HEPA, PTFE (Polytetrafluoroethylene) etc.^23,24^. Each filter layer material is having its own advantages and disadvantages^24^. Among this filter layer material, nonwoven materials are known to be pertinent structures for fine filtration and moderate pressure drop^24^. In order to develop a filter that combines good permeability and high efficiency, polypropylene non woven melt blown are of great interest because of its fine mesh structural parameters, excellent filtration properties, thermal insulation, and sorption capacity and allowing the wearer to breath while reducing the inflow of possible infectious particles^25–27^. Polypropylene (PP) melt blown non woven is the most widely used polymer for this process, since it is relatively inexpensive and versatile enough to produce a wide range of products^25–27^. Others, such as polyethylene (PE), poly(ethyleneterephthalate) (PET), poly(butyleneterephthalate) (PBT), polystyrene (PS), polyurethane (PUR), and polyamide (PA) can also be used to produce melt blown webs^28^. Moreover, non-woven fabric forming technology is cheaper than other fabric forming technology like woven or knitted. Nonwovens are considered as better filter material than woven fabrics owing to its cost effective, cheaper to produce, versatile and offer a wide range of functionalities. Using melt blown technology, hydrophilic and hydrophobic materials can be produced and by incorporating additives it will improve filtration efficiency, moisture adsorption, and biocidal properties^25,26^. It has been observed that in PP-MB-NW, fiber diameter, pore size, and areal density have played a very crucial role on the performance of the filter nonwoven in terms of protective and functional parameters^29^. Hence, the objective of present work is to understand and investigate the filtration efficiency behavior of commercially available different PP-MB-NW which is strongly influenced by its fiber diameter, pore size, pore distribution and air permeability.

Herein, different characteristic properties of PP-MB-NW including pore size, pore diameter, air permeability, filtration efficiency at different aerosol particle size are studied and a relationship was established to its particulate filtration efficiency (PFE). For this purpose, different thickness, areal density, pore size and pore diameters PP-MB-NW are subjected to filtration efficiency test against different aerosol particles. Surface area analysis was carried out for the first time in order to get more insight about the pore having a dimension between 17–3000 Å and total area in pore 11.79 Å which were not possible with porosimeter. PFE of PP-MB-NW were evaluated in single layer and combination thereof against aerosol particulate sizes ranges from 0.3 to 10 μm range, which is particularly relevant for respiratory virus transmission. The PP-MB-NW samples were characterized using scanning electron microscopy (SEM) so as to know the surface morphological characteristics. The study will be imperative as it provides information and understanding about the core layer of respiratory mask, which are keenly looked by the industries or manufacture to explore the right kind of PP-MB-NW fabric to achieve required filtration efficiency.

## Experimental

### Chemical and materials

The polypropylene (PP) melt blown nonwoven fabric of different structural characteristic was provided by the commercial Indian Industry. Sodium chloride purity (≥ 99.0%) was purchased from Sigma-Aldrich, India. Milli-Q water was utilized to prepare sodium chloride solutions.

### Instrumentation and material characterization

ESEM (Make: Carl ZEISS EVO 15 LVSM, Germany) system was used for the surface and structural morphology of the PPE MB nonwoven. The pore size and its distribution were characterized by Mercury porosimeter (Porous Materials Inc, USA). The BET surface area were measured using surface area analyzer (ASAP 2020, Micrometrics, USA). The differential pressure across the PP-MB-NW fabric was measured using pressure meter (Testo 510 absolute Pressure meter, USA). Lighthouse Worldwide Solutions, USA Model No. handheld 2016 was used for the aerosol particle size and concentration. Air permeability of the samples was measured using an Air permeability tester (Make: Asian Equipment, Ghaziabad, India).

### Pressure drop and breathing resistance

Pressure drop was measured by pressure meter through a sample by measuring the difference between downstream and upstream air at constant flow rate. Prior to the experiment, samples are conditioned for a minimum of 24 h by exposure to a temperature of 25 ± 1 °C and a relative humidity of 85 ± 5%^30^. The samples are allowed to return at room temperature for at-least 4 h between exposure and prior to subsequent testing. Breathing resistance was measured by breathing resistance assembly fabricated using IS 9473:2002. The samples were sealed on dummy head. To simulate inhaled air, a vacuum pump pulled air from outside to the inside of the mask at 95 l/min continuous flow of air. Three replicate of each PP-MB-NW filter layer were tested at ambient experimental condition at 95 lpm continuous flow of air using pressure transducer.

### Characterizations of PP-MB-NW fabric

The physical properties of the PP-MB-NW fabric were investigated as per the standard test method. The areal density of the PP-MB-NW was measured by ASTM D 6492-98 and the air permeability was measured by ASTM D 737-04^31^. The solid volume fraction of the PP-MB-NW was calculated using equationn^32^:

1 $$ \alpha \, = 10 \times {\text{G}}/{\text{P}}_{{\text{f}}} \times {\text{Z}} $$

where, G = mass per unit area, P_f_ = fiber density (g/cm^3^), and Z = thickness of the fabric, mm.

Fabric’s areal density (mass per unit area) was measured according to ASTM D3776-09 standard. Each specimen was cut into 5 cm^2^ size using a template and weighed by an electronic balance.

2 $$ \rho = {\text{m/A}} $$

where, ρ is areal density (g/m^2^), m is the mass in g and A is the fabric area in m^2^. Five measurements were conducted for each PP-MB-NW fabric.

### Area of test material and face velocity for particulate filtration efficiency

To determine the particular filtration efficiency of different PP-MB-NW, a specimen of 3.14 cm^2^ area was taken from the PP-MB-NW samples by considering the surface area of a typical facemask as approximately 150 cm^2^ as reported elsewhere^33^. Based on the area of mask, flow rate was calculated for particular specimen and it was 4.5 LPM, representative of 95 LPM. Three samples of each PP-MB-NW were tested for its filter performance. From the mean penetration value, the percentage efficiency was calculated for each melt blown individual and combination thereof.

### Determination of particulate filtration efficiency

**Figure 1:**
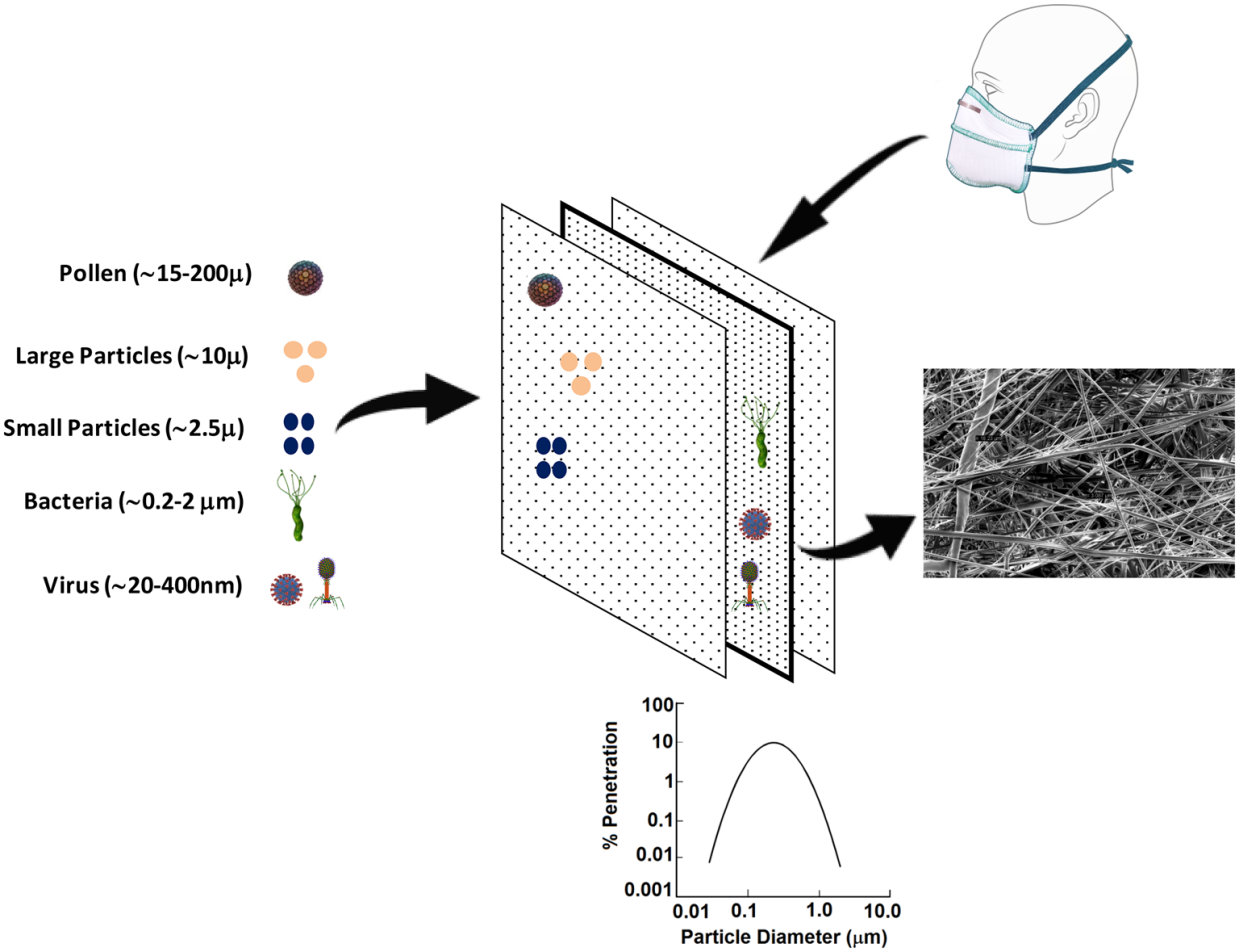
Schematic representation of the respiratory mask showing the various layers and importance of differ layer of mask with emphasis of PP-MB-NW against most penetrating particle size (drawn using Microsoft Office 2007 and Solid Edge version 4).

**Figure 2:**
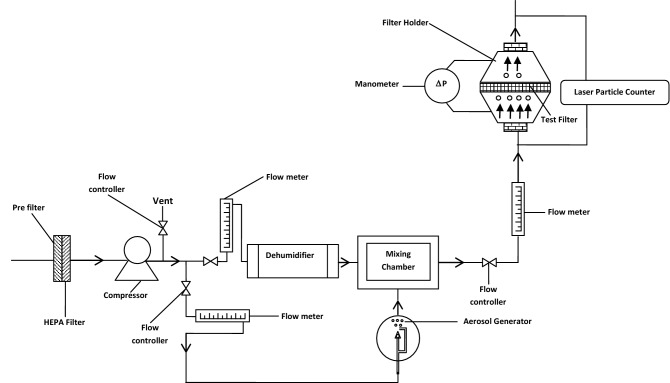
Schematic of the aerosol filtration efficiency test setup (drawn using Microsoft Office 2007).

Figure 1 illustrate the role of various layers of respiratory mask against different particle size with emphasis to core layer i.e. PP-MB-NW against most penetrating particle size of 0.3 µm. The filtration efficiency and pressure drop of PP-MB-NW was tested by using NaCl test rig^34^. The schematic view of the NaCl Test Rig is given in Fig. 2. The test rig consists of the compressor, air receiver, dry air unit, flow meter, control valve, air ducts, aerosol generator, particle counter, and digital manometer etc. as shown in Fig. 2. The dry and clean air was obtained from compressor through HEPA filter and fed to the air regulator. The required airflow is adjusted by means of flow meter and flow control valve. NaCl was used to generate aerosol and the size of aerosol ranging from 0.3 to 10 μm. The test aerosol of NaCl is generated by compressed air atomisation using glass nebuliser. The generator/pump when operated at an air pressure of 1.76 kg cm^2^ (approx) ensures production of NaCl aerosol in the sub micron range with Mass Median Diameter (MMD) of 0.3 μm. The PFE of different PP-MB-NW was measured by laser particle counter by taking the difference of particles from up and down air stream of respiratory mask^35^. The air filtration efficiency is calculated using following equation:

$$ {\text{Particulate}}\;{\text{ filtration}}\;{\text{ efficiency}}\; \, (\% ) \, = \, [({\text{C}}1 - {\text{C}}2)/({\text{C}}1)] \times 100 $$

where C1 is the number of NaCl particle in upstream counts and C2 is the number of NaCl particle in downstream counts.

## Results and discussion

### Influence of structural characteristics of PP-MB-NW fabric

In order to know the effect of structural characteristics of PP-MB-NW fabric on filtration PFE, structural characteristic such as thickness, areal density and solid volume fraction (α) were studied and tabulated in Table 1^17,36,37^. Areal density is an important factor which affects the fluid flow through a nonwoven and it also affects the air permeability^38^. Areal density is also affected by thickness and density of the fibers and finally affects the α^37^. Hence, all these structural parameters are interrelated. As can be seen from the Table 1 the areal density and thickness of the PP-MB-NW samples A–H were varied from 18–60 GSM and 0.08–0.350 mm, respectively. Since, thickness and areal density in combination has ability to affect α or porosity of a fabric. Therefore, α which is also referred as packing density of the fabric are also calculated and showed in Table 1. The α values of samples A–H are calculated and found that it varied from 1980 to 3700. In general, as the areal density increases the α value should also increase, however in this study, regular trend is not observed due to difference in thickness (increase in thickness decreases the α value). However, In sample A and C, the α is at higher side from rest of the samples which reflects the greater degree of compactness of the fabric due to high packing density which could decreases the air permeability and in turn increases the filtration efficiency^39,40^. Hence, from the above data it can infer that the sample A and C may have lower air permeability with high filtration efficiency^41^.

**Table 1:** Structural characteristic of PPE melt blown non woven.

S. no.	Sample code	Thickness (mm)	Aerial density (g/m^2^)	Solid volume fraction (α)
1	A	0.080	25	3703.70
2	B	0.100	28	3094.60
3	C	0.120	30	2818.51
4	D	0.140	30	2412.92
5	E	0.080	18	2584.08
6	F	0.250	50	2332.4
7	G	0.140	30	2412.92
8	H	0.350	60	1983.17

### Influence of structural characteristics of PP-MB-NW fabric

There is limited research on the effect of PP-MB-NW fabrics thickness, solid volume fraction and porosity over air permeability of non woven structure in technical textiles^42^. In general, it has been reported by some of research group that there is a non linear relationship between air permeability and thickness and solid volume fraction^43,44^, though it has also been found that air-permeability is almost inversely proportional to the mass per unit area^45^. However, it is rational to expect that air permeability is strongly dependent on non woven microstructural parameters such as porosity, fiber size, solid volume fraction and fiber arrangement as air permeability governs the air resistance or the pressure drop of the air through fabric^36,46^. In general, respiratory mask is comprised of layers, from outer to inner: an outer hydrophobic layer spun bound followed by 2 melt blown non woven layer (PP-MB-NW) as a filter layer, in between the filter layer, one support layer that provides rigidity and adds thickness to the mask, giving it more structure and adding to the feel of comfort. The innermost layer is another hydrophobic non-woven polypropylene layer which minimizes moisture within the mask from entering the mask material.

**Figure 3:**
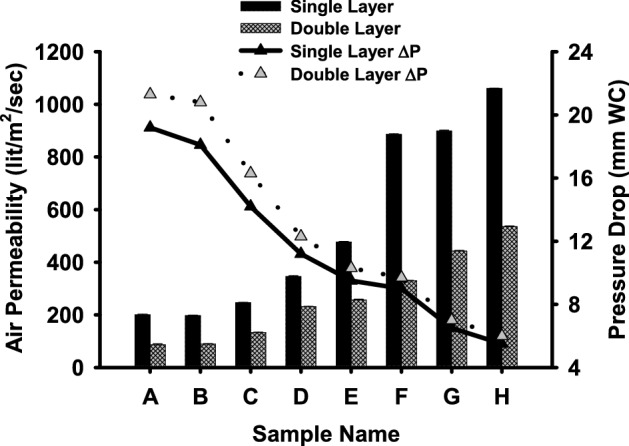
Air permeability and pressure drop of various PP-MB-NW fabric sample (A–H) in single and double layers. The error bars represent the standard deviation of three measurements.

The air permeability and pressure drop of PP-MB-NW samples (A–H) were measured according to ASTM D73^47^. As can be seen from the Fig. 3 and Table 2 the air permeability of sample A to D is varying from 197.24 to 345.84 l/m^2^/s in single PP-MB-NW samples while in combination of double layer it varied from 86.67 to 213.17 l/m^2^/s. While in sample E to H air permeability increased from 477.04 l/m^2^/s to 1060.76 l/m^2^/s in single layer and in double layer it increases from 257.09 to 535.80 l/m^2^/s. As it is observed from Fig. 3, as air permeability increases, pressure drop decreases as it is inversely proportional to the air permeability. This difference in air permeability and pressure drop is due to meltblown technology which enables production of webs having finer fibers with higher surface area, and different degree of interlacing. The decrease in air permeability using 2 melt blown layer is attributed by multilayered structures behaved as a barrier to hinder the flow of air through the structure. The differences in air permeability of the samples are may be due to differences in pore sizes and compactness of these samples. As can been seen from the Table 2, samples A to C are having lower air permeability when compared to rest of the samples and small difference in air permeability of the samples, one can expect that there may be a small variation in pore size, compactness and interlacing in between fibers and may have little variation in PFE^48,49^. The above findings are well supported by solid volume fraction of the various PP-MB-NW samples. As in sample A and B the α value was greater than rest of the samples. In order to get more insight of packing density, fiber diameter, degree of entanglements and distance between the fibers which can greatly influence the air permeability of PP-MB-NW fabrics, it was further characterized for its surface morphology using SEM in subsequent section.

**Table 2:** Air permeability of PPE melt blown non woven samples as per ASTM D73.

S. no.	Sample code	Sample layers	Air permeability @ 12.7 mm WG (l/m^2^/s)
1	A	Single layer	200.24
		Double layer	86.67
2	B	Single layer	197.24
		Double layer	89.33
3	C	Single layer	246.47
		Double layer	132.67
4	D	Single layer	345.84
		Double layer	213.17
5	E	Single layer	477.04
		Double layer	257.09
6	F	Single layer	885.67
		Double layer	329.62
7	G	Single layer	898.51
		Double layer	442.25
8	H	Single layer	1060.76
		Double layer	535.8

### Particulate filtration efficiency of filter layer (PP-MB-NW)

**Figure 4:**
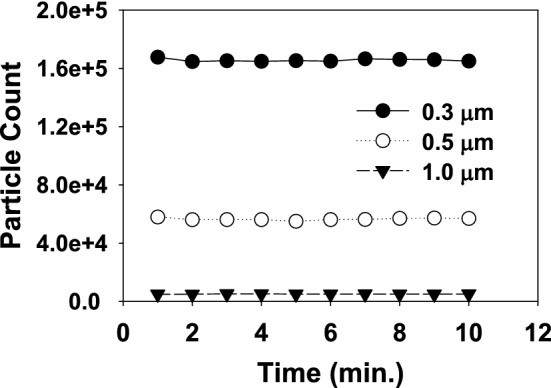
Stability studies of generated NaCl aerosol particle size 0.3, 0.5 and 1 µm.

**Figure 5:**
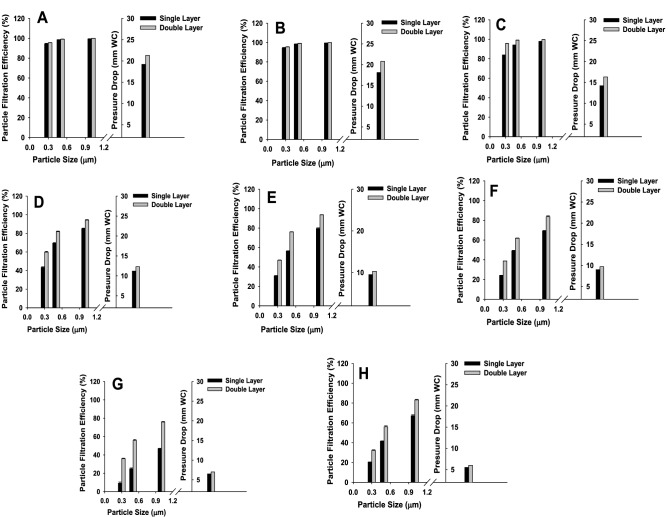
Particulate filtration efficiency and pressure drop of various PP-MB-NW fabric sample (A–H) in single and double layers: Fig. 5A–H: PFE of sample A, B, C, D, E, F, G and H in single and in double layer against NaCl aerosol particle size 0.3, 0.5 and 1 µm. The error bars represent the standard deviation of three measurements.

The filtration efficiency for all the eight PP-MB-NW samples was measured using the NaCl aerosol method as a function of particle size. Prior to the experiment, samples are conditioned for a minimum of 24 h by exposure to a temperature of 25 ± 1 °C and a relative humidity of 85 ± 5%^30^. The samples are allowed to return at room temperature for at-least 4 h between exposure and prior to subsequent testing. The filtration efficiency of the samples has been tested for the particle size of 0.2 µm, 0.3 µm, 0.5 µm and 1 µm. The reason to choose 0.3 µm size particle for PFE is to target the respiratory droplet of Sars-COV-2 coronavirus which is larger than 0.3 µm. The concentration of the upstream and downstream particles of sizes 0.2 µm, 0.3 µm, 0.5 µm and 1 µm were measured using the optical laser particle counter. In order to determine PFE, stabilization of generated aerosol particle size 0.3 µm, 0.5 µm and 1 µm is very important and it was studied for a period of 10 min as given in Fig. 4. As can be seen from the Fig. 4, the generated NaCl particle of different sizes are stable and observed approximately same number of particles. After stabilization of generated aerosol particle, the samples are challenged for three particle sizes of 0.2, 0.3, 0.5 and 1 µm. The aerosol generator can be adjusted to generate the desired concentrations of particle for testing, which are enumerated using the particle counter downstream of the test sample. The aerosol was passed through a drying chamber, diluted to the required concentration using pre filtered air and then passed through the sample. The challenge flow was same as reported in IS 9473:2002. Three replicate of each PP-MB-NW filter layer were tested at ambient experimental condition. The PFE of individual PP-MB-NW layer and their combination was determined as a function of particle size by measuring the upstream concentration and downstream concentration. Table 3 details the results obtained with the individual and its combination of PP-MB-NW layer for PFE, Differential Pressure (∆P) and breathing inhalation resistance (BIR). The ∆P and BIR across PP-MB-NW layer was measured to determine the comfort level of wearer and the suitability of materials to fabricate the mask. Figure 5 and Table 3 shows that, in case of Sample A and B, the PFE of single layer MB against 0.2 μm is 93.37 and 92.62% and against 0.3 μm is 94.10% and 93.06%, respectively, while keeping another layer of MB, the PFE increased to > 99% in both the samples. This observation suggests that, the second layer does not increase PFE significantly; however, the second layer is important to meet the desired PFE as per NIOSH and IS standard. For sample A, the PFE for 0.5 and 1 µm particles are reached against approx 98–99% even in single layer and in combination thereof. Increasing the number of layers (as shown for Fig. 5 and Table 3), as expected, improves the PFE performance. In sample C, the PFE against 0.3 µm particle is 83.94% with single layer and increased to 95.70% using double layer of the same sample. The PFE for size in between 0.5 to 1 µm is varies from 94.06 to 99.09% using single and double layer. The PFE for samples D, E, F, G and H for single layer is 43.37, 30.76, 24.04, 15.5 and 20.41% and it increased to 59.85, 46.94, 38.73, 36.00 and 32.16 for double layer samples, respectively. It has also been observed from Table 3, for 0.2 µm size particle the PFE is approx same as 0.3 µm and similar trend was reported earlier. Higher PFE values were obtained with sample A to C which may be due to highly compact structure of MB with higher degree of entanglement which may lead to decrease in pore dia and distance between fabrics, this results are in agreement with α value of Table 1. Moreover, this wide variation in filtration efficiency for different samples can be attributed by the surface properties of the fibers which play a major role on filtration and the fibers in each layer of non woven fabric and its surface morphology is responsible for filtration performance. The value of ∆P and BIR of the samples are having high PFE are more when compared to samples (D–H) are having a low PFE, nevertheless the values are in the limit of IS 9473:2002. The ∆P and BIR value also support the compactness and high degree of entanglement of PP-MB-NW fabric in sample A–C. In order to further understand the filtration efficiency behavior of different PP-MB-NW samples, pore size, BET surface area analysis and SEM characterization studies were performed and discussed in subsequent sections.

**Table 3:** Particulate filtration efficiency, ∆P and breathing inhalation resistance of PPE melt blown non woven samples.

S. no.	Sample code	Sample layers	PFE (%)				∆P mmWC	Breathing inhalation resistance (BIR) mbar
			0.2 μm	0.3 μm	0.5 μm	1.0 μm		
1	A	Single layer	93.37	94.33	98.44	99.41	19.2	1.85
		Double layer	97.25	98.61	99.13	99.99	21.3	1.92
2	B	Single layer	92.62	93.56	97.50	99.64	18.1	1.72
		Double layer	98.80	99.59	99.95	99.95	20.8	1.88
3	C	Single layer	82.62	83.85	94.08	97.59	14.2	1.42
		Double layer	94.60	95.67	99.08	99.72	16.3	1.52
4	D	Single layer	42.29	43.37	69.43	85.07	11.2	0.89
		Double layer	58.92	59.85	81.81	94.19	12.3	0.95
5	E	Single layer	29.90	30.76	56.21	79.50	9.5	0.69
		Double layer	46.02	46.94	76.04	93.57	10.3	0.73
6	F	Single layer	23.18	24.04	44.05	69.18	9.0	0.55
		Double layer	37.25	38.73	61.87	83.67	9.7	0.58
7	G	Single layer	14.65	15.5	24.84	46.19	6.5	0.54
		Double layer	35.17	36.00	55.75	75.85	7.0	0.57
8	H	Single layer	19.21	20.41	41.46	67.22	5.5	0.44
		Double layer	30.89	32.16	56.00	83.19	6.0	0.51

### Pore size determination using mercury porosimeter

**Figure 6:**
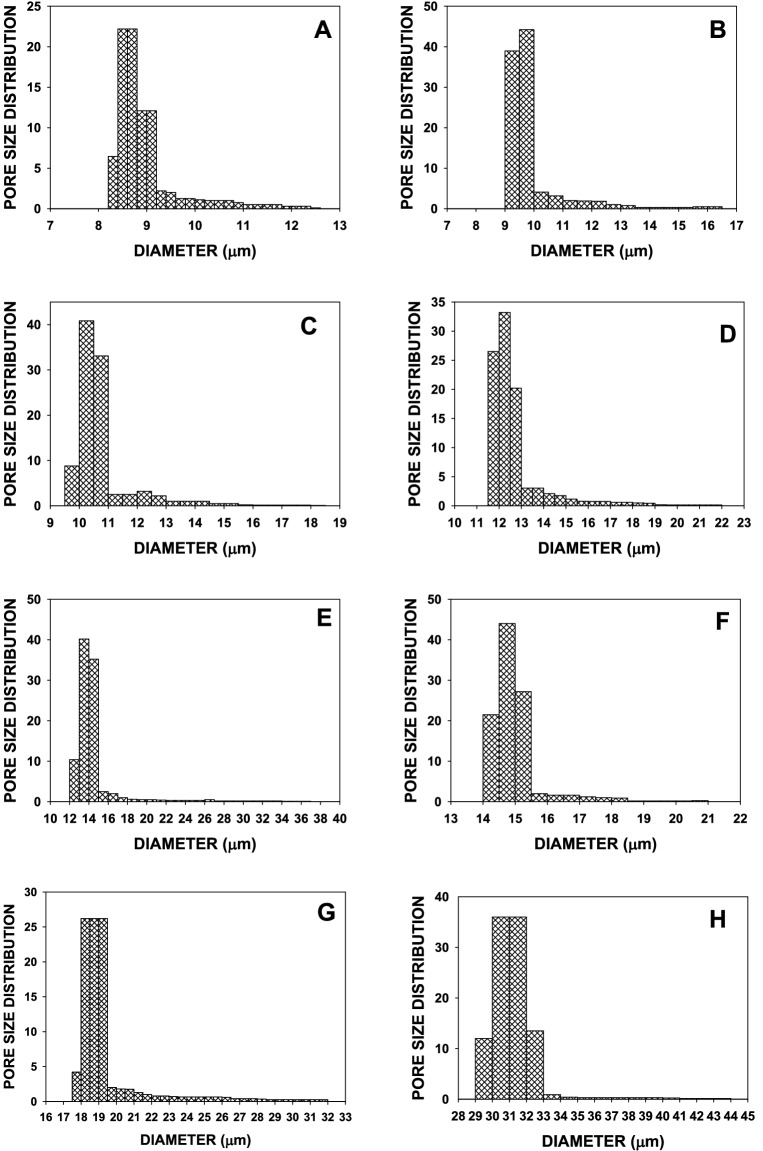
Pore size distribution of different sample (A–H) showing distribution of different diameter pore of the filter layer nonwoven PP-MB-NW fabric. (**A**–**H**) Plot showing pore size distribution vs. pore diameter of sample A, B, C, D, E, F, G and H using mercury porosimeter.

The pore structure and its size plays a very crucial role in performance of PP-MB-NW fabric making this as a most important structural feature^50^. These pore structure is internally connected by three-dimensional network of capillary channels of non-uniform dimensions. In PP-MB-NW fabric fabrics, pore can be classified into three categories namely blind pores, through pores and closed pores. The blind and through pores are playing significant role in PFE. In order to confirm the above finding, mercury porosimeter was used to determine the pore size of different samples^51^. In general pore size decreases due to higher specific surface area of lower fiber diameter^49^. The porosimeter determines both pressure and flow and record these in a pressure versus flow graph for wet and dry sample^51–53^. The dry data curves are determined after all the liquid expelled from the pores. These dry curves become the reference for calculating the pore distribution^51–53^. After measuring the pressure, air flow rate for dry and wet samples, pore size, and pore size distribution are calculated by the software. The results of mercury porosimeter are shown in Fig. 6 and summarized in Table 4.

**Table 4:** Pore size distribution of different MB sample (A–H) showing maximum and mean pore diameter of the filter layer.

S. no.	Sample code	Maximum pore size (µm)	Mean pore size (µm)
1	A	15.92	9.60
2	B	12.49	8.80
3	C	18.65	10.51
4	D	22.32	12.34
5	E	37.81	13.98
6	F	20.81	14.85
7	G	31.95	18.86
8	H	43.80	31.07

Figure 6 shows the curve between pore size distributions versus the pore diameter in order to elucidate the major contribution of pores in filtration efficiency as pore size plays a very crucial role. Table 4 depicts the pore size distribution of various samples of PP-MB-NW fabric in terms of maximum pore size and mean pore size. As can be seen from Fig. 6, in sample A–C, the pore size showed a relatively narrow range, the mean and maximum pore sizes were varied from 8.80 to 10.51 µm and 12.49 to 18.65 μm, respectively. In sample A, the largest peak exists of 9.5–10.0 µm and some major peak between the pore size from 9 to 10 µm, indicating maximum contribution of 9.0–10 µm pore diameter the most to the porosity followed by other pore diameter. In sample B (Fig. 6), largest peak of pore size diameter start from 8.4 to 9 μm, and it contribute most of the porosity in the sample. Likewise in sample C, D, E, F, G and H the most contributing pore diameters are 10–11 µm, 11.5–13.0 µm, 13–15 µm, 14–15.5 µm, 18–19.5 µm and 30–32 µm , respectively. As can be seen from the above, there is an increasing trend in pore diameter from sample A to H and similar tends are also observed in the air permeability from sample A–H. This increment in mean pore size was also supported by solid volume fraction and air permeability data shown in Tables 1 and 2, respectively. Furthermore, PFE behaviors of different PP-MB-NW samples (A–H) are also supported by the above data, as fiber diameter plays a significant effect on filtration efficiency properties. Low fiber diameter leads to better filtration efficiency owing to higher entanglement and higher surface area as observed in case of samples A–C. This data also confirmed that the air permeability is inversely proportional to mean pore dia of the nonwoven PP, as reported elsewhere^38^. Moreover, the difference in maximum pore size and mean pore size of the samples are attributed by the influence of sample material, surface morphology, operation process and the use of wetting agent^54^.

### BET surface area characterization of PP-MB-NW

Using mercury porosimeter, the through pore diameter can be only measured up to the range of micron size only. However, the smaller pore diameter including blind pore cannot be characterized by utilizing mercury porosimeter as these pores are not accessible by the fluid to flow through them. Hence, the size of blind pore cannot be determined. In order to understand the PFE behavior sometime porosimeter and air permeability data along with microstructures details above are not sufficient enough to characterize the MB for its behavior. Hence, BET surface area analyzer was used to characterize and overcome the above limitation of porosimeter as blinds pores are also responsible for filtration efficiency. In order to understand and to get more insight in particular, the pore sizes which are below the limit of porosimeter, pore having a dimension between 17–3000 Å and total area in pore 11.79 Å were calculated. As can be seen from the Table 5, the sample A and B the total area of pores 11.79 Å are more than 20 m^2^/g with BJH desorption Cumulative volume of pores between 17.0 Å and 3000 Å width approx more than 0.031 cm^3^/g are having good filtration efficiency. The resultant data from above characterization (role of structural characteristic, air permeability, through and open pore, and surface morphology of MB fibers) collectively contribute on the PFE of PP-MB-NW fabrics.

**Table 5:** Surface area analyzer study of MB sample (A–H) for the determination of open pore at one end having a dimension between 17–3000 Å and total area in pore 11.79 Å.

S. no.	Sample code	Total area in pores 11.79 Å	BJH desorption Cumulative volume of pores between 17.0 Å and 3000 Å width
1	A	20.967	0.04332
2	B	20.36	0.082
3	C	13.397	0.060
4	D	10.93	0.031
5	E	6.852	0.016
6	F	4.50	0.008
7	G	1.93	0.00168
8	H	2.90	0.00542

### SEM characterization of PP-MB-NW fabric samples

**Figure 7:**
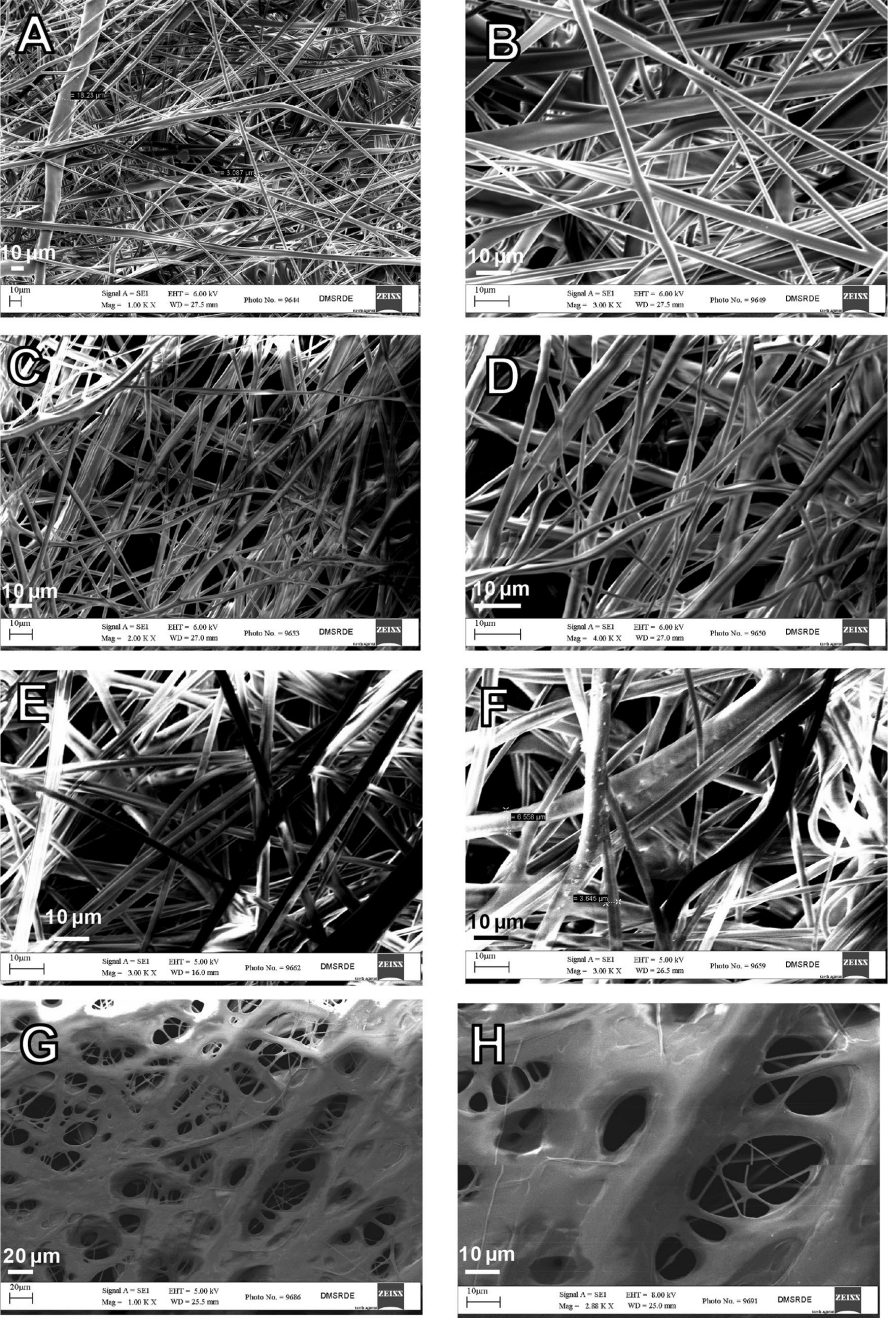
SEM images of the microscopic structure of various PP-MB-NW of sample A–H based on PFE (**A**,**B**) PFE: 90–99, (**C**,**D**) 70–90, (**E**,**F**) 50–60 and (**G**,**H**) 30–20.

Analysis of microstructure is important to explain the above finding of PP-MB-NW materials; hence scanning electron microscopy (SEM) characterization studies were performed. Figure 7, displays the SEM images of the PP-MB-NW fabrics. This characterization studies were performed to investigate the surface morphology, the fiber diameter and density of entanglement. Since, 16 SEM images were taken for 8 samples (A–H), however, it is found that there is apparently no difference in the structural morphology of samples which are having approximately same PFE. Hence, the total samples A–H have been divided in to 4 categories based on PFE (i) PFE: 90–99 (Fig. 7A,B), (ii) 70–90 (Fig. 7C,D), (iii) 50–60 (Fig. 7E,F) and (iv) 20–30 (Fig. 7G,H), and only representative figure are presented here. As can be seen from the Fig. 7A,B (magnified image) which belong PFE to the range of 90–99, the rod shaped fibrous structures with non-uniform morphology was clearly observed with a wide range of diameters varied from 1 to 10 μm of PP-MB-NW fibers with a high degree of entanglement with each other. The diameters of individual fibers in a web appear to change along the length of the fibers. The fiber junctions were also visible which was produced from thermal sticking, interlacing and branching of MB fibers. Compactness between the fibers decreases as we move down from sample C to H. The fibers entanglement in sample A, B and C are more when compared to rest of the sample which reflects the decrease in pore size of fibers due to entanglement^49^. Furthermore, in case of larger diameter fibers, the chances of entanglement are relatively lower and this contributes larger distances between fibers and also leads to larger diameter fibers^49^. As can be seen from Fig. 7G,H (magnified image) which belong to filtration efficiency of 20–30%, less fibers per unit area which increase the distances between fibers. Though the fiber diameter is less in Fig. 7G,H as compared to rest of the sample, yet PFE is low due to low and wide spinneret orifice distributions which lead to increase the distance between the fibers during the manufacturing. Hence, it can be concluded from SEM images that PP-MB-NW fabrics which are having high entanglement, high fibers per unit area and the reduced distance between the fibers lead to higher filtration efficiency and this data support the findings of PFE and air permeability^29–55^.

### Effect of charge on filtration efficiency of PP-MB-NW

**Figure 8:**
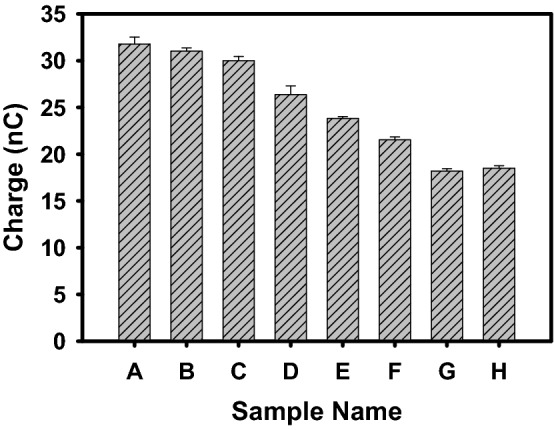
Charge of various PP-MB-NW of sample A–H (calculated using Standard EN 1149-1).

In order to find out the effect of PP-MB-NW charge on filtration efficiency, resistance of sample A to H were measured utilizing Agilent 4339B, USA high resistance meter. The charge of various samples was calculated using resistance data. The charges of PP-MB-NW were shown in Fig. 8 and tabulated in Table 6. From the Table 6 and Fig. 8, it can be observed that there is a qualitative correlation between charge and filtration efficiency. From sample A to C the charge ranges from 30 to 31.78 nC which gives more than 95% PFE, while in samples D to H the decremented trend is observed for both charge and PFE. Hence, from the above data it can be inferred that higher the surface charge on PP-MB-NW will lead to higher PFE. However, this result is only qualitative in nature and only using this parameter it is difficult to conclude about the PFE. Therefore, PFE largely depends on the cumulative effect of microstructural parameters, electrostatic charge, air permeability, diameter of through pore, availability of closed pore etc.

**Table 6:** A comparison between filtration efficiency and charge of PP MB_NW samples A–H.

Sample code	Charge (nC)	Filtration efficiency (%)
A	31.7800	98.61
B	31.0200	99.51
C	30.0000	95.67
D	26.3700	59.85
E	23.8200	46.94
F	21.5300	38.73
G	18.2000	36.00
H	18.5000	32.16

## Conclusions

This study brings a useful insight to the selection of appropriate PP-MB-NW fabrics as effective air filter media, on the basis of their microstructural parameters such as porosity, pore diameter, fiber size, solid volume fraction, fiber arrangements, air permeability and pore size distribution. The filtration efficiency of different PP-MB-NW fabric samples is investigated in single and double layers combination for the particle size of 0.3, 0.5 and 1 µm. Surface area analysis was done for the first time in order to get more insight about the blind pore having a dimension between 17–3000 Å and total area in pore 11.79 Å which were not possible with mercury porosimeter and found that the PP-MB-NW fabrics having total area in pores 11.79 Å more than 10 m^2^/g with BJH desorption Cumulative volume of pores between 17.0 Å and 3000 Å width approx more than 0.031 cm^3^/g is exhibiting good filtration efficiency. The samples which are having the mean and maximum pore sizes in the range from 8.80 to 10.51 µm and 12.49 to 18.65 μm, respectively are having a good filtration efficiency. From the above data, it can be inferred that the PP-MB-NW fabrics those are having low pore dia, high solid fraction volume, low air permeability are having high filtration efficiency against 0.3 µm particle which reflects the importance of these structural parameters on PFE. Based on only one microstructural parameter, it is difficult to judge the performance of respiratory mask. However, the cumulative effect of these microstructural parameters which are interconnected with each other governs the performance of the PP-MB-NW fabrics in terms of filtration efficiency. We believe that this study will be very useful as it provides information and understanding about the core layer of respiratory mask, and provide a directive for the judicial selection of filter material to achieve high filtration efficiency to combat COVID-19.
